# Traffic planning in modern large cities Paris and Istanbul

**DOI:** 10.1038/s41598-024-64483-w

**Published:** 2024-06-15

**Authors:** Yunus Emre Ayözen, Hakan İnaç

**Affiliations:** 1 Ministerial Advisor, Ministry of Transport and Infrastructure, Ankara, Turkey; 2 Investment Management & Control Department, Directorate for Strategy Development, Ministry of Transport and Infrastructure, Ankara, Turkey

**Keywords:** Direction, Flow, Model prediction, Traffic planning, Velocity, Civil engineering, Engineering

## Abstract

The enhancement of flexibility, energy efficiency, and environmental friendliness constitutes a widely acknowledged trend in the development of urban infrastructure. The proliferation of various types of transportation vehicles exacerbates the complexity of traffic regulation. Intelligent transportation systems, leveraging real-time traffic status prediction technologies, such as velocity estimation, emerge as viable solutions for the efficacious management and control of urban road networks. The objective of this project is to address the complex task of increasing accuracy in predicting traffic conditions on a big scale using deep learning techniques. To accomplish the objective of the study, the historical traffic data of Paris and Istanbul within a certain timeframe were used, considering the impact of variables such as speed, traffic volume, and direction. Specifically, traffic movie clips based on 2 years of real-world data for the two cities were utilized. The movies were generated with HERE data derived from over 100 billion GPS (Global Positioning System) probe points collected from a substantial fleet of automobiles. The model presented by us, unlike the majority of previous ones, takes into account the cumulative impact of speed, flow, and direction. The developed model showed better results compared to the well-known models, in particular, in comparison with the SR-ResNet model. The pixel-wise MAE (mean absolute error) values for Paris and Istanbul were 4.299 and 3.884 respectively, compared to 4.551 and 3.993 for SR-ResNET. Thus, the created model demonstrated the possibilities for further enhancing the accuracy and efficacy of intelligent transportation systems, particularly in large urban centres, thereby facilitating heightened safety, energy efficiency, and convenience for road users. The obtained results will be useful for local policymakers responsible for infrastructure development planning, as well as for specialists and researchers in the field. Future research should investigate how to incorporate more sources of information, in particular previous information from physical traffic flow models, information about weather conditions, etc. into the deep learning framework, as well as further increasing of the throughput capacity and reducing processing time.

## Introduction

Unprecedented levels of urbanisation have caused cities to grow in both size and population. Improved traffic state prediction accuracy can help organizations and politicians better understand the fundamental mechanisms that control urban traffic, which will help them create more logical transportation regulations^1^. This will facilitate a reduction in the number of accidents and violations, as well as minimize the required time for passenger and freight transportation, yielding significant economic and environmental advantages in terms of resource conservation and reduction of harmful emissions^2,3^.

The Intelligent Transportation System (ITS) has demonstrated its effectiveness in improving traffic efficiency, alleviating congestion, and promoting overall traffic development as urban traffic continues to increase^4^. In addition to flexible traffic regulation, intelligent transportation systems also have the capability to expand with additional functionalities and capabilities, such as automatic notification of relevant services in case of emergencies, warning drivers of sudden obstacles on the road, more sophisticated detection of traffic violations, and so forth. This collectively forms a complex progressive system that renders the city safer and more efficient. Traffic flow prediction, being a primary focus of Intelligent Transportation Systems (ITS), has garnered increasing interest^5^. The inherent unpredictability of the traffic flow poses challenges to the process of prediction^6^. Hence, the process of acquiring knowledge about randomness via artificial intelligence algorithms should be given careful thought^7^.

The majority of studies that are now available are direct implementations of machine learning techniques without a full examination of the dynamics of traffic conditions based on domain expertise and physical interpretations. An important trend in traffic research is the synthesis of multidisciplinary knowledge and experience, which is especially useful for creating powerful deep-learning models. Furthermore, these studies sometimes only consider a small portion of a city rather than the full region in high resolution, which limits the utility of their practical applications. Satisfactory prediction of the current state of traffic in the city is essential to maintain its sustainability.

The objective of this project is to address the complex task of predicting traffic conditions on a big scale using deep learning techniques. For the purpose of conducting the research, data pertaining to traffic in two cities—Paris and Istanbul—were selected. Paris has an official estimated population of 2,102,650 residents as of 1 January 2023 in an area of more than 105 km^2^. Paris is the 30th most densely populated city in the world in 2022. It is also characterised by rather poor traffic conditions, being, according to the TomTom Traffic Index report for the year 2023, the 16th worst in terms of traffic conditions out of 387 analysed cities, with an average congestion loss of 120 h per road user per year. This indicator demonstrates a negative trend in recent years, despite active initiatives in the city aimed at addressing traffic issues, such as the construction of bus lanes and bicycle paths, and efforts to limit transit traffic, which contributes to half of the congestion. Among the reasons cited for the problem are insufficient parking spaces, the closure of the central road Voie Georges-Pompidou for environmental reasons, and inefficient planning, whereby an increase in the number of roads instead of reducing congestion leads to a decrease in overall traffic efficiency (known as the Braess paradox)^8^. Istanbul is the largest city in Turkey, has a population of over 15 million residents, comprising 19% of the population of Turkiye. Istanbul is the most populous European city and the world's 15th-largest city. The metrics for Istanbul, as per the same TomTom Traffic Index report, are more favourable, ranking 65th with an average time loss of 104 h per year. Although this indicator shows some improvement, the city's position in the ranking still underscores the necessity for further efforts. Issues exacerbating traffic conditions in Istanbul include poor road planning, underutilisation of maritime routes, inadequate coordination among traffic systems, and suboptimal timing of traffic signals. Both cities are major metropolises; however, their conditions and challenges vary significantly. Paris is situated along crucial land transit routes, whereas Istanbul is a significant port city. Testing the model in cities with such diverse conditions will validate its adaptability and capacity to address varying needs^9,10^.

To accomplish this objective, it is important to analyse the historical traffic data of Paris and Istanbul within a certain timeframe, considering the impact of variables such as speed, traffic volume, and direction. The industrial scale traffic status data will be given by The Institute of Advanced Research in Artificial Intelligence (IARAI). Particularly, there included information regarding traffic movie clips based on 2 years of real-world data for Paris and Istanbul. The movies will generate data derived from over 100 billion GPS (Global Positioning System) probe points collected from a substantial fleet of automobiles. For the investigations, the authors will employ used next samples and models: information from the same time last week; previous time slice information; a basic model; a model, which supports velocity from earlier in the day; model is fed previous traffic conditions in close time slices rather than periodic data; model, is created by feeding multi-source data. The developed model will use three levels (velocity, flow, and direction) showed better results compared to the well-known models. The resultant model should be compared to the established models employed for traffic prediction.

## Literature review

The way traffic conditions change over time demonstrates how travel demand changes. This behaviour was originally modelled early in the last century, although it was simplified owing to a lack of data^11^. So far, one of the most well-known traffic forecasting methods has been the method that used an autoregressive integrated moving average. However, this method has several disadvantages, especially taking into account seasonal deviations^1–7,12^. A different line of study, incorporating activity-based travel demand models, developed the traffic forecast problem based on traffic assignment in addition to the autoregressive integrated moving average^13,14^. Due to the growing popularity of machine learning, the topic is best phrased as a supervised learning problem. Support vector machines^15^, k-nearest neighbours^16^, extreme value count models^17^, and stacked autoencoders^18^ were used to capture the underlying nonlinear interactions. Recent studies have looked closely at the relationships between the spatial and temporal dimensions, which are frequently supported by new deep-learning approaches^19–21^.

The paper^22^ presents a new approach that combines deep learning algorithms to accurately predict the current traffic volume at three neighbouring crossings. An analysis of raw traffic volume data involves utilising a two-layer network consisting of the gated recurrent unit (GRU) and long short-term memory (LSTM) models. Additionally, the wavelet transform (WL) noise reduction strategy (WL + GRU-LSTM) was implemented. The WL + GRU-LSTM model was created following a thorough analysis of several machine learning and deep learning techniques. A comparative study was conducted to select the network topology, training technique, and optimizer type for the model. In order to demonstrate the model's precision and durability, it was tested against the top methods for forecasting immediate traffic conditions. The experimental findings validate the capability of the WL + GRU-LSTM model to accurately predict intricate variations in traffic volume across various intersection configurations, attaining an accuracy rate of 94%.

Given its success in the field of computer vision, the convolutional neural network is frequently utilized in traffic prediction applications to handle spatial correlations^23–25^. By stacking traffic maps as separate channels, it may also describe temporal patterns. A convolutional structure, for instance, was used by Zhang et al.^24^ to capture the spatial dependency between areas. To address the temporal linkages, traffic maps arranged as channels in various time slices were used. Guo et al.^26^ used 3D convolutions to directly address the temporal dimension rather than employing channels. Dai et al.^27^ were able to reorder those pixels according to correlation coefficients, which is different from many convolutional neural network-based algorithms that produce traffic maps based on the physical position of sensors. To describe the temporal dynamics of traffic, Ke et al.^28^ and Ma et al.^29^ merged CNN (Convolutional neural network) with RNN (recurrent neural network). Yao et al.^23^ divided complicated traffic patterns into three viewpoints, with CNN handling the geographical information, RNN (such as a long short-term memory network) handling the temporal information, and structure embedding extracting the semantic dependence. A square grid form is also better suited for data like photographs than urban road networks, according to some academics. Some have also claimed that graph-based models can better describe the topological structure of the road network^30,31^. Diffusion convolutions were used to determine how traffic flow is dependent on space in the initial use of graph modelling for traffic prediction^32^.

Rathore et al.^33^ developed an original method for controlling traffic in large cities under conditions of varying visibility. For this purpose, moving car cameras were used, as well as a video processing module developed based on a special graphics processor. The system has shown its effectiveness in terms of increasing throughput and reducing processing time.

To predict traffic, Yu et al.^34^ also used the graph convolution technique. A sequential combination of graph convolution and GRU (gated recurrent unit) was suggested by Zhao et al.^35^ In a study by Lin et al.^36^, an algorithm was proposed for predicting the demand for bicycles, taking into account the connection between docking stations. Additionally, a graph filter was created to take station heterogeneity into account. Cui et al.^37^ suggested using the flexible graph wavelet technique to extract local spatial patterns for traffic prediction. This would enhance common graph convolutional neural networks. The graph convolution architecture suggested by Zhang et al.^38^ includes an attention mechanism to evaluate the applicability of input data for traffic velocity prediction.

The research regions of many excellent publications on spatiotemporal prediction only cover a limited fraction of a city due to data availability limitations^23,39,40^. Data granularity has an impact on spatiotemporal data. The information loss in coarse data might have a very detrimental effect on model performance. The research region is often divided into the low-resolution picture in studies on spatiotemporal data prediction that provide state-of-the-art performance. As an illustration, Zhang et al.^40^ arranged two cities into a grid map with a few subregions, each measuring 1 km^2^. However, with such a resolution, it is difficult to identify the intersections and linkages. Fine-grained information must thus be preserved since it is crucial. A smaller subregion's size allows for easier maintenance of the network structure.

The subsequent sections of this study are organised as follows. The Methods and Materials are defined in "Methods and materials", and the dataset is then presented. The Results and Discussion are described in "Results" and "Discussion" accordingly. Finally, "Conclusion" provides the Conclusions.

## Methods and materials

### Data description

The industrial scale traffic status data are given by IARAI (The Institute of Advanced Research in Artificial Intelligence)^41^. Particularly, there included information regarding traffic movie clips based on 2 years of real-world data for Paris and Istanbul. The movies were generated with HERE Technologies data derived from over 100 billion GPS probe points collected from a substantial fleet of automobiles. The data has undergone complete anonymization and has been converted into high-definition movie clips that accurately portray traffic patterns over time, including morning, evening, and peak hour traffic incidents, frame by frame. The files provide historical traffic information for two cities in 2021, Paris (France) and Istanbul (Turkiye) (Table 1).

**Table 1:** The dataset description.

Dataset	Istanbul	Paris
Traffic data type	Velocity, flow, direction	
Length of time slice, min	5	
Size of map	436 × 495	
Spatiotemporal cell size, m	100 × 100	
Study area coverage (km^2^)	2158.2	
Kind of weather	100	
Duration of constant weather conditions (min)	30	

**Figure 1:**
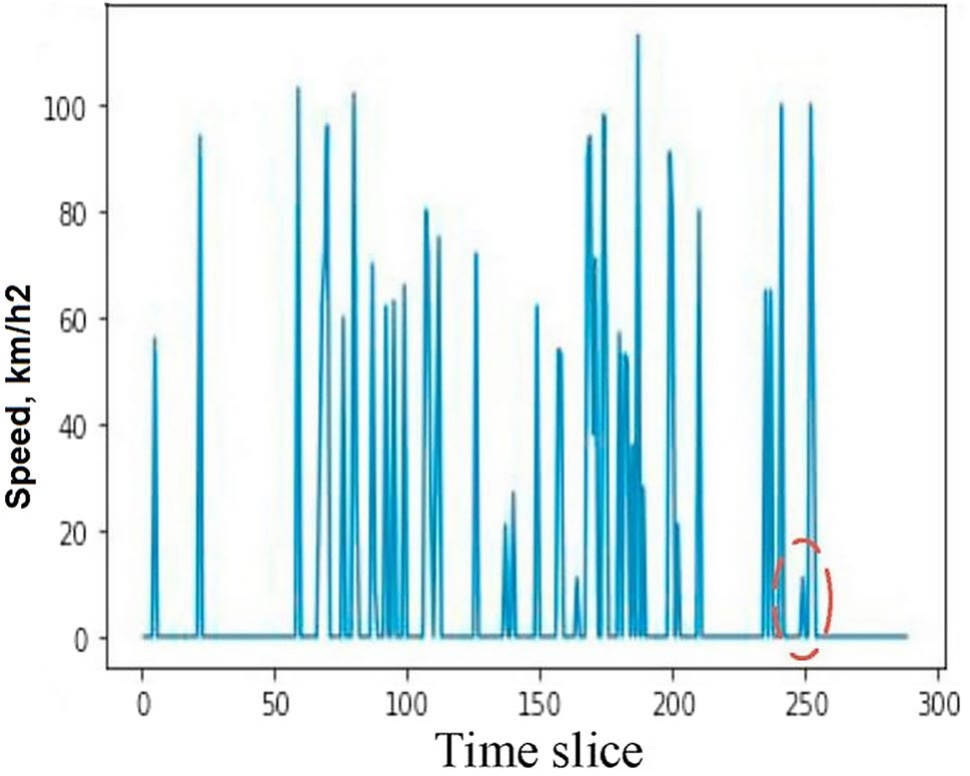
Time slice of average velocity on a day in Paris.

**Figure 2:**
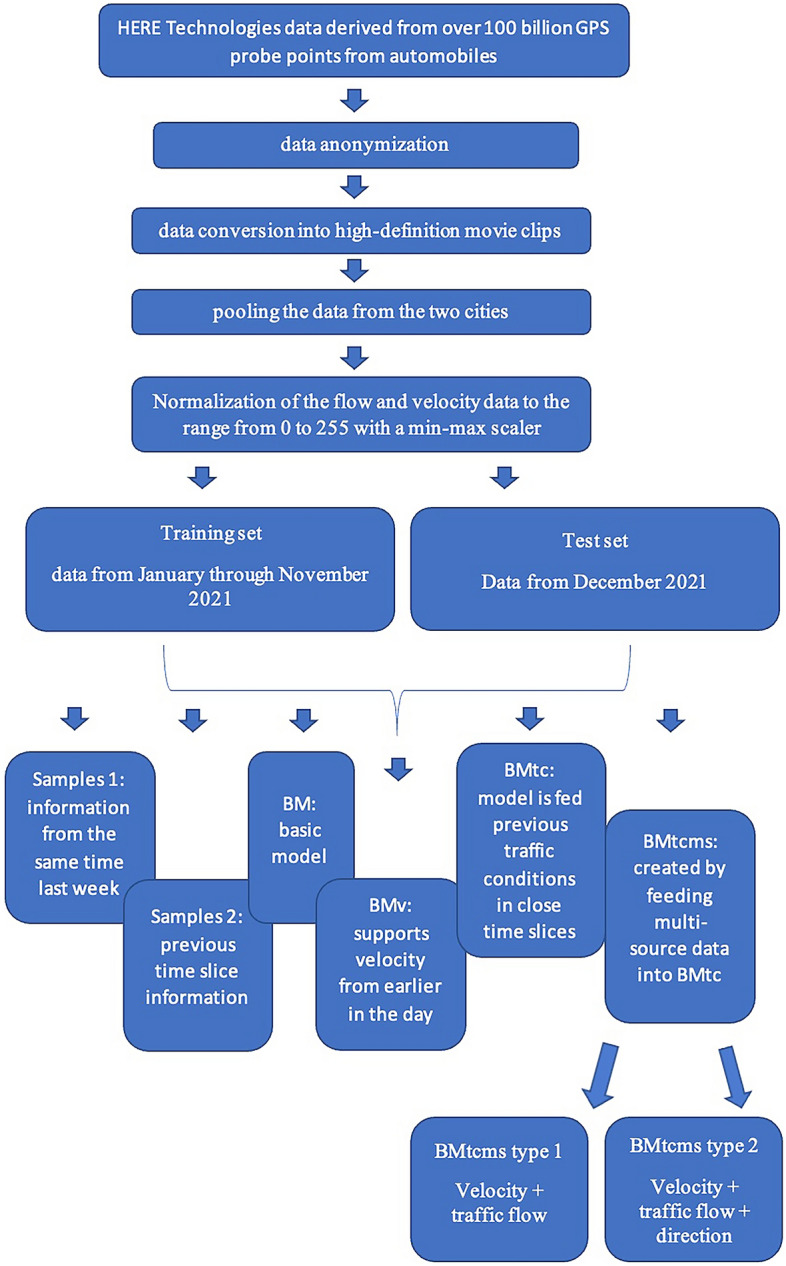
The overall flow chart of the research steps.

Three channels, one each for the traffic flow, velocity, and direction, are present in every frame. To build a traffic map of 436 × 495 pixels and a 2000 km^2^ region, the data from each city are pooled. It keeps track of traffic data across a region of 100 square meters for each pixel. The data are combined on a 5-min basis, or 288 frames in total, every day. Additionally, a min–max scaler was used to normalise the flow and velocity data to the range from 0 to 255. The directions of the probes that were launched from the north pole are recorded in the data along with their heading angles, which are binned into four digits, which stand for northeast (1), southeast (85), southwest (170), and northwest (255), respectively. On a traffic map, each pixel, or so-called spatiotemporal cell, represents a subregion of p square meters for the city as a whole. The recorded trajectories from a sizable fleet of probe vehicles over a year were used to create the traffic map. Each frame is sampled once every q minutes. Tensor ${S}_{d}^{t}$ can be used to represent the velocity in all spatiotemporal cells during the *t*-th time slice of the *d*-th day. Data from time series on traffic are often continuous rather than discrete by nature. An example time series plot of the average velocity variations on a day in Paris is shown in Fig. 1. The overall flow chart on the research steps is presented in Fig. 2.

### Mathematical description

**Figure 3:**
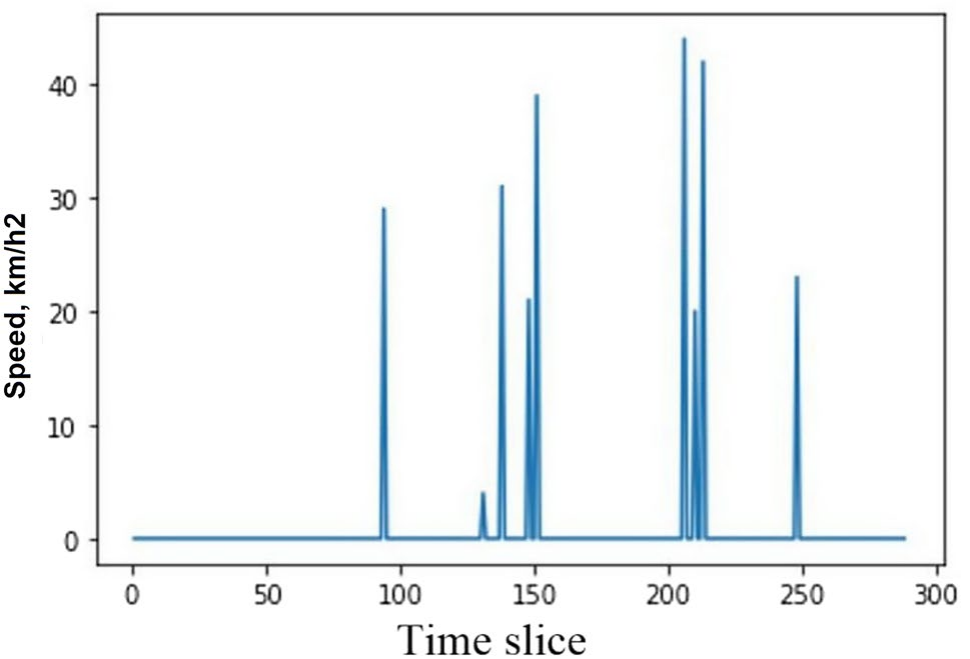
Time slice of velocity on a day at a pixel in Paris.

As a result of the strong similarity between the traffic states at different time slices, temporal dependence is typically a crucial factor in time series prediction. Therefore, velocity at *t*-1, *t*-2,…, and *t*-*k* will be handled as input characteristics, represented as $[{S}_{d}^{t-1},{S}_{d}^{t-2},\dots ,{S}_{d}^{t-k}]$, to forecast the velocity at time *t* of day *d*, designated by ${S}_{d}^{t}$. However, only a few time slices, as shown in Fig. 3, have velocity records at a pixel.

According to the study, after *k* time slices, the traffic state would spread to other subregions over the linkages of the road network. To predict the city-wide velocity at a certain point in time, images obtained at different points in time must be included in the input feature map. It should be noted that even though they are shown as $[{S}_{d}^{t-1},{S}_{d}^{t-2},\dots ,{S}_{d}^{t-k}]$, they represent the propagation of traffic state.

The following study on congestion propagation is offered to simplify comprehension of how traffic situations spread. In the network, if node *a* encounters traffic congestion, it will propagate upstream and produce congestion in nearby nodes. According to the theory of traffic flow, one must determine the flow in each direction at node *a* to indicate which node will experience congestion soon. Furthermore, the potential time for the congestion may be determined by taking into account the distances between node *a* and neighbouring nodes.

The model will learn how states spread by replaying past traffic states in narrow time slices. The model can use previous learnings if the traffic status is shown to spread up here in historical data. Additionally, the traffic conditions from the previous *m* days may be utilized to recreate the experience and are represented as follows:

1 $$\left[{S}_{d-1}^{t-k},{S}_{d-1}^{t+k}\right]\cup \left[{S}_{d-2}^{t-k},{S}_{d-2}^{t+k}\right]\cdot \cdot \cdot \cup \left[{S}_{d-m}^{t-k},{S}_{d-m}^{t+k}\right].$$

Keep in mind that there are (2 × k + 1) × m input channels. There will be too many channels to input when *m* and *k* are big, necessitating additional compression. For instance, average global pooling operations may be used to compute the average velocity of 2 × k + 1 time slices to compress traffic states in the *m*-th day for $[{S}_{d-m}^{t-k},{S}_{d-m}^{t+k}]$ along the channel dimension. Then, from 2 × k + 1 to 1, the number of channels will be decreased. The following formulation describes this procedure:

2 $${S}_{d-m}^{t}=globa{l}_{pooling\left(\left[{S}_{d-m}^{t-k},{S}_{d-m}^{t+k}\right]\right)},$$

where the compressed traffic states are represented by ${S}_{d-m}^{t}$:

3 $$\left[{S}_{d-1}^{t}, {S}_{d-2}^{t},\dots , {S}_{d-m}^{t}\right].$$

It is commonly believed that combining information from several sources might aid in traffic prediction. The authors used external data, such as weather, holidays, days of the week, and times of day, to inform the model. The external characteristics are included in the x-dimensional vector and added to the U-Net through two entirely connected layers. In addition to the external data, the forecast of velocity is aided by the use of traffic flow $[{F}_{d}^{t-1},{F}_{d}^{t-2},\dots ,{F}_{d}^{t-k}]$ and direction $[{D}_{d}^{t-1},{D}_{d}^{t-2},\dots ,{D}_{d}^{t-k}]$, which are two additional sources of data. The traffic flow and direction during the last m days in k consecutive time slices will be used for experience replay, as indicated below.

4 $$\left[{F}_{d-1}^{t-k},{F}_{d-1}^{t+k}\right]\cup \left[{F}_{d-2}^{t-k},{F}_{d-2}^{t+k}\right]\cup \cdots \cup \left[{F}_{d-m}^{t-k},{F}_{d-m}^{t+k}\right],$$

5 $$\left[{D}_{d-1}^{t-k},{D}_{d-1}^{t+k}\right]\cup \left[{D}_{d-2}^{t-k},{D}_{d-2}^{t+k}\right]\cup \cdots \cup \left[{D}_{d-m}^{t-k},{D}_{d-m}^{t+k}\right].$$

The data for each day are then compressed into a single channel and represented as $[{\underline{F}}_{d-1}^{t},{\underline{F}}_{d-2}^{t},\dots ,{\underline{F}}_{d-m}^{t}]$ and $[{\underline{D}}_{d-1}^{t},{\underline{D}}_{d-2}^{t},\dots ,{\underline{D}}_{d-m}^{t}]$. There will be 14 × 3 channels when the data from the past two weeks are utilized. In feature map *M*, roads include pixels with values greater than zero; for this reason, these pixels are assigned the number 1. The mask is offered as stated below.

6 $${mask}_{i,j}=\{1, if {M}_{i,j}>0, 0, if {M}_{i,j}=0,$$

where ${M}_{i,j}$ stands for the pixel ($i,j$) value. The mask is layered to the input channels of U-Net and suggests the structure of the road network.

Practitioners in many ITS (Intelligent Transportation System) applications are frequently more focused on variables that are easy to comprehend. As a consequence, the pixel-wise MAE (mean absolute error) is used to evaluate the performance of the model.

7 $$MAE=\frac{1}{m}{\sum }_{i=1}^{m}\left|{S}^{i}-{\widehat{S}}^{i}\right|,$$

where ${S}^{i}$ is the observed speed during the *i*-th time slice, ${\widehat{S}}^{i}$ is the predicted values, and *m* is the total number of predicted samples.

In statistics, mean absolute error (MAE) is a measure of errors between paired observations expressing the same phenomenon. Examples of Y versus X include comparisons of predicted versus observed, subsequent time versus initial time, and one technique of measurement versus an alternative technique of measurement.

For the investigations, the authors used the next samples and models^42^:

- Samples 1: information from the same time last week;
- Samples 2: previous time slice information;
- BM: basic model;
- BM_v_: model, which supports velocity from earlier in the day;
- BM_tc_: model is fed previous traffic conditions in close time slices rather than periodic data;
- BM_tcms_: is created by feeding multi-source data into BM_tc_.

The full input channels to the BM may be written as:

8 $$\begin{aligned} & Input = concat([S_{d}^{{t - 1}} ,S_{d}^{{t - 2}} , \ldots ,S_{d}^{{t - k}} ],[F_{d}^{{t - 1}} ,F_{d}^{{t - 2}} , \ldots ,F_{d}^{{t - k}} ], \\ & [D_{d}^{{t - 1}} ,D_{d}^{{t - 2}} , \ldots ,D_{d}^{{t - k}} ],[S_{{d - 1}}^{t} ,S_{{d - 2}}^{t} , \ldots ,S_{{d - m}}^{t} ],[F_{{d - 1}}^{t} ,F_{{d - 2}}^{t} , \ldots ,F_{{d - m}}^{t} ], \\ & [D_{{d - 1}}^{t} ,D_{{d - 2}}^{t} , \ldots ,D_{{d - m}}^{t} ]), \\ \end{aligned}$$

where *concat*() denotes the joining of smaller tensors to create a tensor with a greater dimension.

## Results

The authors established the velocity between 08.00–10.00 and 17.00–20.00 h as the objectives since urban road management has to pay greater attention to traffic conditions during peak hours^43^. The training set is made up of data from January through November 2021, while the test set is made up of data from December 2021.

Since velocity is the main concern and most of the previous research did not take into account the combined impacts of velocity, flow, and direction, the authors solely examined the modules of this framework using the information on velocity in Table 1. As a consequence, the performances of various variations are evaluated in comparison to the subsequent two baselines, with the findings displayed in Table 2. Each time slice in this study's traffic map has over 200 thousand pixels due to its size.

**Table 2:** Mean absolute error.

Model	Istanbul	Paris
Sample 1	6.254	6.950
Sample 2	5.728	6.395
BM	3.998	4.490
BM_v_	3.966	4.460
BM_tc_	3.917	4.421
BM_tcms_	3.913	4.417

Table 2 shows that utilizing only historical data will not produce good results. All suggested model versions have errors that are much lower than those of Sample 1 and Sample 2. On the datasets for Paris and Istanbul, the mean absolute error of the fundamental model (BM) is 4.490 and 3.998 respectively. The use of BM_v_ was added to 4.460 and 3.966, which marginally enhanced the findings of the core model on two datasets. The model BM_tc_ shows a greater improvement to the fundamental model, attaining the mean absolute error of 4.421 and 3.917 for the two cities, respectively. Finally, the authors tried using auxiliary multi-source data, such as meteorological data, but no appreciable increase was seen: 4.417 and 3.913 for Paris and Istanbul, respectively. This is considered to be related to the size of the research region and the availability of supplemental data sources. Numerous monitoring stations are frequently dispersed across a megacity that covers thousands of square kilometres since the weather in different areas of a city might vary greatly. Unfortunately, one of these stations contributed to the meteorological data used in the dataset, which makes it unrepresentative of the whole city. Additionally, this task's prediction horizon is 5 min. This necessitates a quick weather update as well.

Liang et al.^44^ used a grid map with a very fine-grained resolution of 64 × 64 pixels to divide their research area. Their goal was to super-resolve a given map to infer the fine-grained crowd movement rather than predict the future^44^. A unique model that allows the connection of road characteristics was created by Pan et al.^39^ The graph-based models, as indicated, significantly rely on trustworthy base maps. As a result, the following well-known generic models are used to assess the performance of the proposed model.

One of the most recent models for spatiotemporal prediction is ST-ResNet^25^, which is utilized in comparison with the suggested framework. Grid maps with sizes of 32 × 32 and 16 × 8 are used to split the two datasets that ST-ResNet uses. Additionally, because of the poor resolution, there are no issues with sparsity or apparent periodicity. The authors also add components to the model that deal with traffic flow and vehicle direction. Due to the three aforementioned obstacles, it is important to note that this task is substantially more complex than the conventional spatiotemporal forecast of urban traffic.

Based on BM_tcms_, which simply makes use of velocity information, two variations are constructed. The first type makes use of data on velocity and traffic flow, whereas the second variant also incorporates direction data. Table 3 compares the resulting model to a cutting-edge deep learning model.

**Table 3:** Mean absolute error for different models.

Model	Istanbul	Paris
SR-ResNet	3.993	4.551
BM (velocity)	3.913	4.417
BM (velocity, flow)	3.890	4.405
BM (velocity, flow, direction)	3.884	4.299

Table 3 shows that the accuracy increased when the flow was included in BM (velocity), or the BM_tcms_ in Table 2, to 4.405 and 3.890. Further, the MAE was enhanced to 4.299 and 3.884 (the data which aid in supporting road forecast). Although limited by the receptive field, ST-ResNet (velocity, flow, direction) is also capable of properly predicting the traffic status (4.551 and 3.993 for Paris and Istanbul, respectively). Table 4 displays the results of some settings (*k* and *m*).

**Table 4:** Mean absolute errors for different parameters.

Setting		Istanbul	Paris
*k*	*m*		
12	7	3.898	4.315
12	14	3.884	4.299
12	21	3.887	4.310
24	14	3.898	4.303

If one week's worth of traffic states are repeated, the value of *m* will equal 7. The prediction performance is not always improved by larger *k* and *m*, as the increased number of channels might result in a significant computing burden and the new characteristics may be redundant. The most suitable are parameters for *k* = 12 and *m* = 14.

The study's replicability lies in its transparent methodology and use of publicly available real-world data. Researchers can replicate the methodology by accessing similar traffic datasets, applying the described deep learning models, and following the outlined preprocessing steps. Additionally, the clear description of model variations, parameter optimization, and evaluation metrics facilitates reproducibility. Collaboration with institutions like IARAI for access to similar data or collaboration with HERE Technologies for similar GPS probe datasets can further support replicability. Overall, adherence to the described methodology, access to relevant data sources, and transparent reporting enable researchers to replicate and validate the study's findings effectively.

## Discussion

Recognising the status of traffic is crucial for intelligent transportation systems and is important for developing strategies to reduce congestion and provide real-time assistance for vehicles. The road operation information may be gathered by utilising the traffic information gathering equipment installed on the road. The traffic condition can be inferred based on the data from traffic flow operations.

It is necessary to compare the database used in this work with the databases that were used in similar works. Table 5 displays a comparison between this dataset with various other spatiotemporal prediction datasets.

**Table 5:** Datasets.

Data type	Taxi^14^	Bike^14^	Yao et al.^13^	Liu et al.^31^	The current work
Length of time slice (min)	30	60	30	30	5
Size of map	32 × 32	16 × 8	20 × 20	25 × 50	436 × 495
Pixel, number	1024	128	400	1250	215,820
Size (m)	1000 × 1000	1000 × 1000	700 × 700	10 × 10	100 × 100
Study area coverage (km^2^)	1024	128	196	0.125	2158.2

The report by Zhang et al.^24^ contained the first mention of TaxiBJ and BikeNYC. Yao et al.^23^ tested their model using the Didichuxing traffic dataset. Liang et al.^44^ used data from Happy Valley to do their study on crowd flow. Such an indicator as Time slice length in the current work was only 5 min, in contrast to other works, in which it ranged from 0.5 to 1 h. The traffic map size in the present work was also the maximum and amounted to 436.495, in contrast to other works, where it amounted to such values as 32.32; 16.8; 20.20; and 25.50. It should also be noted that such an indicator as the number of pixels in the present work was 215,820 and in other works 1024; 128; 400 and 1250. The comprehension of the intricate urban transportation system that exists across the city can be greatly aided by such large-scale experiments. Furthermore, to reduce the dependency on road network data, the authors employ the grid format.

This study aimed to solve the deep learning-based challenge of large-scale traffic status prediction. The authors achieved this goal, by studying the historical data on traffic in Paris and Istanbul in a certain period, taking into account the influence of such factors as velocity, flow density and direction. Based on the data obtained, the authors developed a traffic forecast model for these cities. The resulting model was compared with the known models used to predict traffic. The developed model using three levels (velocity, flow, and direction) showed better results compared to the well-known SR-ResNet model. The MAE values for Paris and Istanbul were 4.551 and 3.993 for SR-ResNET and 4.299 and 3.884 for the developed model, respectively.

## Conclusion

Forecasting traffic in large cities is one of the main problems of the normal functioning of various life support systems in these cities. Intelligent transportation systems will facilitate reductions in energy resource expenditures through traffic optimization and congestion alleviation, a decrease in the number of accidents, and further promote the proliferation of environmentally friendly practices such as vehicle sharing. The effective functioning of such practices relies heavily on prediction and optimization mechanisms. To ensure the beneficial impact of such systems, continuous attention to ensuring their accuracy and expanding capabilities is imperative.

The objective of this project is to address the complex task of predicting traffic conditions on a big scale using deep learning techniques. To accomplish this objective, the historical traffic data of Paris and Istanbul within a certain timeframe were analyzed, considering the impact of variables such as speed, traffic volume, and direction. The results of the experiment showed the effectiveness of the developed technique in forecasting large-scale traffic situations. The developed model, which used three levels (velocity, flow, and direction) showed better results compared to the well-known SR-ResNet model. The MAE values for Paris and Istanbul were 4.551 and 3.993 for SR-ResNET and 4.299 and 3.884 for the developed model, respectively. Thus, the obtained result will aid in addressing the complexities of traffic regulation in heavily congested metropolitan areas, where this issue is most pressing. The obtained results can be utilised in the development of more precise and efficient intelligent transportation systems for large cities, as well as in further research endeavours aimed at enhancing the performance metrics of such systems. It is recommended for local policymakers accountable for city infrastructure development to pay attention to new findings in the area of the intelligent transportation systems, including the current study. Paying attention to the last developments in the field can suggest planning and implementing new projects for development more safe and efficient city infrastructure, as well as encourage investment in further research and training of specialists in the field.

Understanding the numerous limitations of the study (as well as any subsequent research) is crucial. First, the study used data from two sizable cities with a spatial resolution of 100 × 100 m and a temporal resolution of 5 min. It would be fascinating to look into how the suggested model functions in medium and small cities, as well as at various geographical and temporal dimensions. However, more explicit analyses of the underlying geographic factors, such as land use, distance to the city centre, layout of the road and transit network, and the nature of the terrain are still lacking. Such regional factors might enhance a prediction model's "transferability". Also, in this study, it was not possible to fully use all potentially valuable information for traffic prediction, in particular, there were problems with the analysis of weather conditions. Since the proposed model is in the process of development, further testing is also necessary to fully identify possible sources of inaccuracies or errors.

It is sensible to continue the endeavour of broadening the spectrum of various types of information that can be effectively utilised by models for improved forecasting, including weather conditions, events that may impact traffic, and so forth. Further efforts in expanding the spatial coverage of intelligent transportation systems, increasing their throughput capacity, and reducing processing time remain pertinent. Future research should investigate how to incorporate previous information from physical traffic flow models into the deep learning framework. It is worthwhile to look at ways to better incorporate this knowledge of these physical rules into data-driven models while maintaining computing efficiency.

## Data Availability

Data will be available from the corresponding author Yunus Emre Ayözen or Hakan İnaç on request.
